# Management of severe liver injuries: push, pack, pringle – and plug!

**DOI:** 10.1186/s13049-021-00907-0

**Published:** 2021-07-14

**Authors:** Arezo Kanani, Knut Olav Sandve, Kjetil Søreide

**Affiliations:** 1 Department of Gastrointestinal Surgery, Stavanger University Hospital, Stavanger, Norway; 2 Department of Radiology, Interventional Radiology Unit, Stavanger University Hospital, Stavanger, Norway; 3 Stavanger Medical Image Laboratory, Stavanger University Hospital, Stavanger, Norway; 4 Department of Clinical Medicine, University of Bergen, Bergen, Norway

**Keywords:** Trauma, Liver injury, Angioembolization, Resuscitation, Bleeding, Damage control

“*Rupture of the liver is fortunately an accident not often met with, but one which, when it is seen, may be associated with a condition of the patient as serious as anyone can meet within surgical practice*” [1].

These are the introductory words of the article by the Glasgow surgeon J. Hogart Pringle, written over a century ago, that would describe the clinical and experimental attempt at surgical control of severe liver lacerations by means of clamping the vessels in the hepatoduodenal ligament [1] – a procedure since referred to by the eponymous “Pringle’s manoeuvre” (Fig. 1). Notably, Pringle himself noted in his seminal paper that in severe cases of bleeding the only right thing to do was to pack. Hence, the surgical dogma for managing severe bleeding of the liver has been to ***Push*** (to approximate the rough wound-edges towards each other for compression), ***Pack*** (to ensure tight packing and compression of the liver parenchyma) and ***Pringle*** (to temporize and reduce the inflow of the portal vein and hepatic artery to the liver). This surgical dogma has been taught for decades, yet with high mortality rates for patients suffering severe hepatic lacerations and bleeding. However, since the emergence and widespread use of cross-sectional imaging by computed tomography in the 1980s and 1990s, the emergence of non-operative management has slowly and steadily increased for all solid organ injuries [2–4]. Added to this is the increased understanding of injury pathophysiology and haemodynamics with appropriate resuscitation measures [5, 6], better organized trauma care and structured team training [7], and added tools and novel workflow to control haemorrhage in selected patients as an adjunct to surgery or, even increasingly, as the only intervention needed [8–10]. Indeed, these adjunct techniques and principles are what constitutes the backbone of successful non-operative management for many trauma patients [3]. Alas, one size does not fit all, and for some patients the best treatment is surgery for timely and proper control of bleeding.

**Fig. 1:**
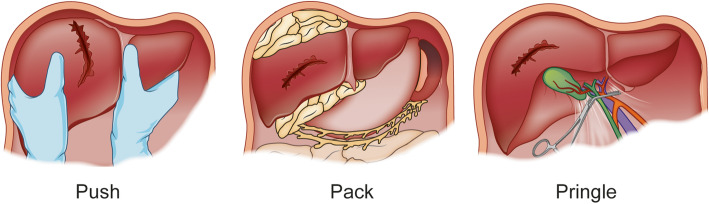
Surgical management of severe bleeding from a liver injury. Legend: The damage control surgical principles of “push, pack and Pringle” to stop liver haemorrhage during surgery

## Adding the “plug” to the toolbox

One of the most important advances over the past couple of decades is the use of angioembolization to control bleeding in severe liver injuries [11, 12]. While the role of interventional radiology continues to expand in trauma care, there is still scarce data on patients presenting with signs of hemodynamic instability and the outcome of this intervention. Thus, the report [13] in this issue of the Journal is timely and of interest to the wider readership. In their study Tamura and colleagues presented an observational, retrospective study enrolled patients with severe blunt liver injury (e.g. grades III–V with American Association for the Surgery of Trauma Organ Injury Scale; AAST-OIS) with and without hemodynamic instability. They included 62 patients, of which most (*n* = 54) underwent angioembolization (8 had operative management alone). The injury severity of the population is indicated by high injury severity scores (mean of 26), the overall mortality (6%) and that only one-third were hemodynamically stable. Despite the widespread differences between groups and the variation in the data they present [13], the authors conclude that angioembolization in hemodynamically unstable patients who had some respond to initial infusion therapy has acceptable in-hospital mortality and clinical failure rates. Of note, the association is not causation, and no causality can be drawn from such a retrospective, observational cohort. The inherited bias in treatment selection is present, as is in the very nature of the design of such cohorts. However, there may be value in the data for others to learn from.

### Defining the liver injury severity – a moving target

Liver trauma represents one of the most common life-threatening abdominal injuries. The liver is a large organ with high blood flow and severe damage to the liver my cause severe bleeding that can be fatal. Understanding different treatment modalities in these patients is of importance to reduce morbidity and mortality. Hence, the dynamic situation starts with the ABC and monitoring of vitals, where the very sick patient should be taken to the operating theatre for control of any suspected massive ongoing hemorrhage. Early initiation of damage control resuscitation, massive transfusion protocol and damage control surgery is essential.

Liver injuries can be defined by their anatomical description [14] based on imaging, operative findings or on pathology [15] or, better yet as attempted by the World Society of Emergency Surgery (WSES) by incorporating the hemodynamic status [16]. Comparing likes with likes is still difficult. Indeed, liver trauma classification as defined by WSES [16] uses four categories, while organ injury scale use six categories. These categories are combined into a three-level grading of liver injury in the WSES guidelines [16] into minor, moderate and severe.

**Fig. 2:**
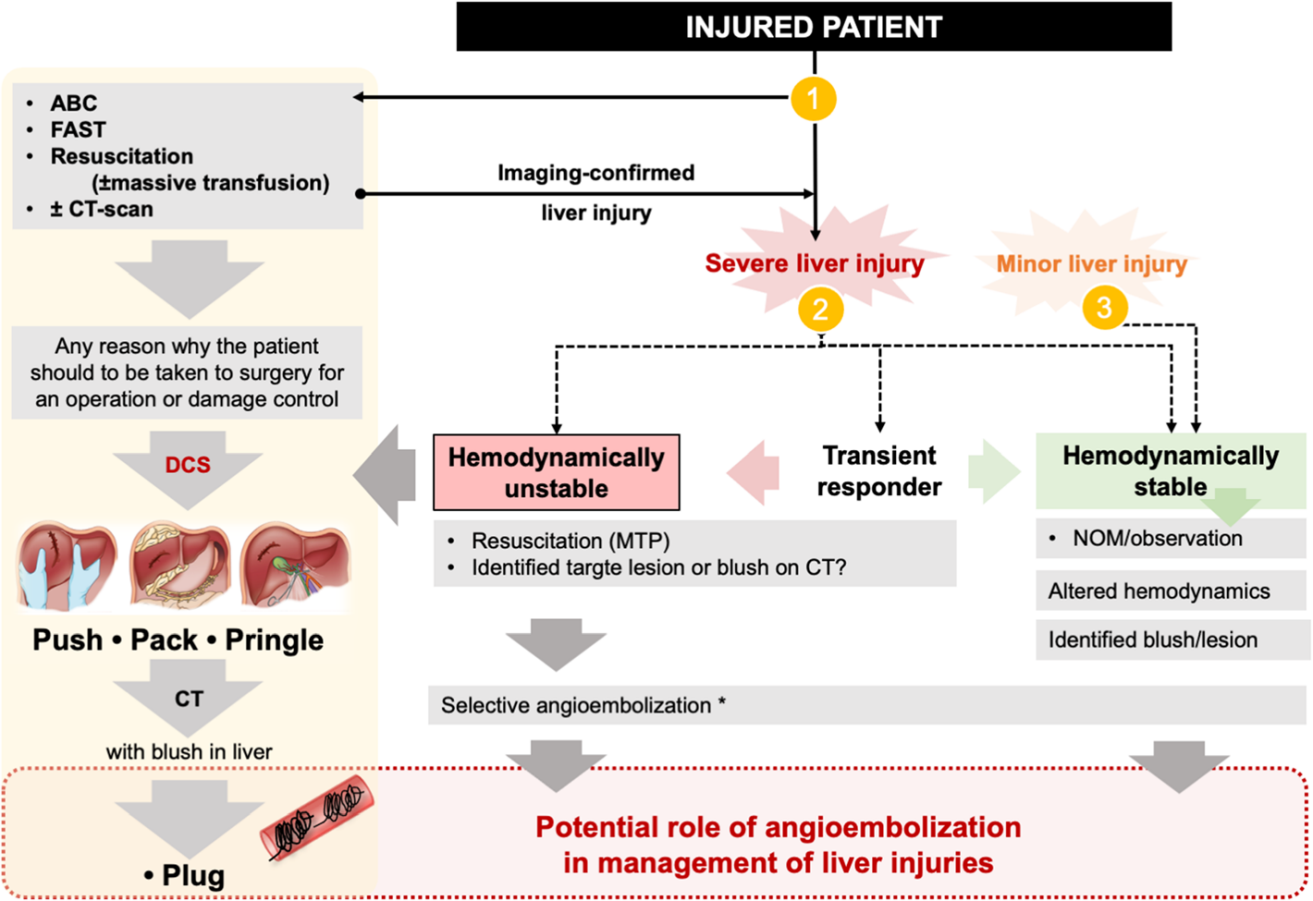
Flow chart showing anticipated role of angioembolization in liver trauma. Legend: The illustration is intended as a rough diagram of clinical scenarios involving liver injury. Indicated is the evaluation of an injured patient that (1) may undergo initial work-up only to proceed directly to damage control measures, e.g. due to ongoing or suspected massive hemorrhage. If imaging can be obtained (2) a situation of a severe liver injury can be declared, with a potential specific operative plan (also, including damage control surgery; DCS). If responding to transfusion and sequential monitoring is in place, a plan for angioembolization may be considered, knowing that higher grade liver injuries are associated with higher failure-rate of non-operative treatment (NOM). In the case of (3) minor liver injury, angioembolization may still have a role in select cases and may be associated with higher success rates for NOM. Detailed algorithms are provided in the existing guidelines, as referenced in the text

Liver injuries can be treated either non-operatively [16–18] or operatively [16, 19]. Three main factors are used to differentiate which line of treatment should be initiated: hemodynamic stability, concomitant injuries and, patients’ age (e.g. children ≤16 years are considered somewhat differently; not discussed here as beyond the scope if this article). Angioembolization may have a role in any or more of the treatment strategies for a given patient (Fig. 2). Knowledge of the strength and limitations to each is paramount to achieve an optimal outcome.

### Where does angioembolization fit in?

Most of the current guidelines on management of severe liver trauma and their subsequent revisions and updates [16–20] have incorporated interventional radiology in some way or another, while there may be nuances and variations across recommendations given (Fig. 2). Notably, while angioembolization has added favourably to the overall success of non-operative management [8, 9], there are also pitfalls to consider [21]. One is the failure of non-operative management despite angioembolization. Hence, clinical vigilance must always be exercised in the team and the interventional radiologist should know the limits [22]. Also, there are a number of potential complications that may develop from angioembolization as well. As the clinical use of angioembolization develops in clinical trauma care it is valuable to document, record and analyse real-life data from such practice.

## Adjunct role of angioembolization to non-operative management of severe liver injury

Non-operative management (NOM) is recommended for all hemodynamic stable patients regardless of type of liver injury and without other injuries requiring surgery including local exploration of wound in the abdominal wall which confirms an intact anterior fascia. Patients in shock in transient response may be considered for NOM provided that continuously close monitoring is available during resuscitation with transfusion and angioembolization with the possibility of rapid transport to an operating room if the patient’s condition deteriorates. In some cases where there is doubt in CT findings or patients clinical condition, diagnostic laparoscopy can give further information. Non-operative management is reported to be successful in up to 85% of minor to moderate liver injuries. However, this success rate rapidly drops with increasing severity of the liver injury and concomitant burden of trauma, in addition to hemodynamic parameters and their dynamics.

## Adjunct role of angioembolization to operative management

OM is recommended in all hemodynamic instable patients or in patients where NOM is ineffective. Primary goal of operative management is to control the haemorrhage and bile leak. Depending on the degree of injury, haemorrhage can be controlled by simple measures like compression, electrocautery, coagulation, topical haemostatic agents or simple suture. In more serious bleeding, hepatic packing, ligation of vessels, Pringle’s manoeuvre, hepatic resection can be attempted, with the latter option mainly when absolute necessary to obtain haemorrhagic control. In case of excessive life-threatening haemorrhage, the placement of resuscitative endovascular balloon occlusion of the aorta (REBOA) can be lifesaving and be a bridge to definitive haemorrhagic control can be obtained. For local haemorrhage control from the liver, Pringles manoeuvre (clamping of portal vein, hepatic artery and choledochus), can be performed until the problem is localized and fixed. Clamping should not exceed 20–60 min to avoid excessive ischemia times, particularly in the hypotensive, shocked patient. However, necessary means to stop bleeding must be prioritized. If there still is uncontrolled, bleeding from liver despite Pringle’s manoeuvre there might be an aberrant hepatic artery present or the bleeding can be extrahepatic from hepatic vein or vena cava. After DCS, a new CT scan (or, if proceeding directly to the OR without CT scan) may be obtained. If properly packed, most venous bleeding should be controlled. However, arterial injury may still produce a blush, and angioembolization of arterial source of bleeding may then be pursued for hemorrhage control. A strategy of selective or supra-selective embolization techniques to the injured vessels may be used [23, 24], but more centrally located or named vessels should be avoided for total occlusion and subsequent ischemia. Given the dual supply by the liver (portal and arterial), even extensive embolization may be well tolerated. Late sequelae in form of biliary leaks, parenchymal necrosis and abcesses are reported.

## Targeted role of angioembolization to specific injury to liver or its vessels

If there is damage to proper hepatic artery it is preferable to try and repair the injury. If repair fails to give haemorrhagic control, ligation can be performed, including cholecystectomy due to risk of necrosis if the right or common hepatic artery must be ligated. It is even more crucial to try and repair injury to the portal vein main branch since ligation of the vein proximally can lead to liver necrosis or massive bowel oedema. If portal vein ligation is inevitable, one must ensure that hepatic artery is intact to ensure blood perfusion to the liver. Interventional radiology access to arterial vessels can facilitate the use of stents rather than coils, to allow for maintained perfusion while stopping a bleed. Liver lobe resection can be performed if the bleeding is related to intraparenchymal vessels that cannot be sutured. If the bleeding is extrahepatic liver packing will often be successful if direct repair of the injured vein is unsuccessful.

Angioembolization has gained an increasing role in the management of severe liver injuries. Careful selection and knowledge of the benefits and limitations of the added tool in the trauma team is a prerequisite for its best use and for optimal patient-centered outcomes.
